# Vitamin D and Clinical Outcomes in Head and Neck Cancer: A Systematic Review

**DOI:** 10.3390/nu17071100

**Published:** 2025-03-21

**Authors:** Cristian Ion Mot, Delia Ioana Horhat, Nicolae Constantin Balica, Bogdan Hirtie, Norberth-Istvan Varga, Catalin Prodan-Barbulescu, Alexandru Alexandru, Elena Ciurariu, Radu Galis

**Affiliations:** 1 Ear, Nose, and Throat Department, “Victor Babes” University of Medicine and Pharmacy Timisoara, Eftimie Murgu Square No. 2, 300041 Timisoara, Romania; mot.ion@gmail.com (C.I.M.); horhat.ioana@umft.ro (D.I.H.); balica@umft.ro (N.C.B.); 2 Doctoral School, “Victor Babes” University of Medicine and Pharmacy Timisoara, Eftimie Murgu Square No. 2, 300041 Timisoara, Romania; norberth.varga@umft.ro (N.-I.V.); catalin.prodan-barbulescu@umft.ro (C.P.-B.); 3 Department of General Medicine, “Victor Babes” University of Medicine and Pharmacy, Eftimie Murgu Square 2, 300041 Timisoara, Romania; alexandru.alexandru@student.umft.ro; 4 Physiology Discipline, Department of Functional Sciences, Faculty of Medicine, “Victor Babeș” University of Medicine and Pharmacy Timisoara, Eftimie Murgu Square No. 2, 300041 Timisoara, Romania; ciurariu.elena@umft.ro; 5 Department of Medical Sciences, Faculty of Medicine and Pharmacy, Oradea University, 410087 Oradea, Romania; rgalis@uoradea.ro

**Keywords:** vitamin D, head and neck cancer, HNC, clinical outcomes, vitamin D cancer, vitamin D receptor

## Abstract

**Background/Objectives:** Vitamin D is classically associated with calcium and phosphate homeostasis, but recent research has expanded its role to include several new roles such as immune regulation, inflammation, and potential anti-cancer properties. The vitamin D receptor (VDR) is expressed in over 400 tissues, including those of the head and neck, implying a potential link between vitamin D and head and neck cancers (HNCs). Given the need for newer and better therapeutic approaches, this systematic review aims to synthesize existing clinical evidence on the relationship between vitamin D status and clinical outcomes in HNC patients. **Methods and Results**: A comprehensive literature search, across multiple databases including PubMed, Google Scholar and Science Direct, identified 187,642 studies related to vitamin D and cancer, from which 16 studies met the inclusion criteria. The inclusion criteria were English-language, full-text original research (2015–2025) on vitamin D’s role in HNC progression and treatment, focusing on human studies. The findings indicate that vitamin D deficiency is highly prevalent among HNC patients, with rates ranging from 47% to 95%, particularly in advanced-stage cancers and those undergoing intensive treatment. Inverse association between vitamin D levels and HNC risk was reported, with higher serum 25(OH)D levels linked to a 30–32% reduction in cancer risk. Additionally, higher vitamin D levels correlated with improved survival rates and reduced recurrence, though some findings lacked statistical significance. Deficiencies were associated with higher rates of malnutrition and postoperative complications, reinforcing vitamin D’s role in nutritional stability and surgical recovery. **Conclusions**: This systematic review highlights how common and significant vitamin D deficiency is among head and neck cancer (HNC) patients, exploring its possible role in cancer risk, prognosis, survival, treatment-related side effects, malnutrition, and post-surgical complications. The evidence suggests that while higher vitamin D levels are linked to better survival and fewer treatment-related issues, the benefits seem to level off beyond a certain point, indicating a more complex relationship. Additionally, vitamin D supplementation appears to help reduce chemoradiation side effects like mucositis, skin toxicity, dysphagia, and pain, ultimately improving patients’ quality of life during treatment.

## 1. Introduction

Vitamin D, a fat-soluble secosteroid, has long been recognized for its pivotal role in maintaining calcium and phosphate homeostasis, which are essential for bone mineralization and skeletal integrity [1,2]. It is synthesized in the skin via ultraviolet B (UVB) exposure to 7-dehydrocholesterol, which turns into pre-vitamin D that spontaneously converts to cholecalciferol (commonly known as vitamin D3), which can also be ingested through diet and supplements. Cholecalciferol undergoes two hydroxylation steps, the first in the liver to form calcidiol (25-hydroxyvitamin D, 25(OH)D), which is the primary circulating form and marker of vitamin D status, and the second in the kidneys to produce calcitriol (1,25-dihydroxyvitamin D, 1,25(OH)2D), its biologically active metabolite [3,4,5]. Transported by vitamin D-binding protein (DBP) in plasma, calcitriol exerts its effects by binding to the vitamin D receptor (VDR), a nuclear transcription factor expressed in numerous tissues [6]. Beyond skeletal health, recent decades have unveiled vitamin D’s broader physiological roles, including immune modulation, inflammation regulation, and cellular differentiation, sparking interest in its potential as an anti-cancer agent [7,8,9,10]. These discoveries stem from VDR’s presence in over 36 organs and hundreds of cell types, ranging from immune cells like lymphocytes to epithelial tissues, suggesting a systemic influence far beyond its classical domain.

This expanded understanding has fueled research into vitamin D’s association with cancer, a disease marked by uncontrolled cellular proliferation and immune evasion. Epidemiological studies have linked low serum 25(OH)D levels to increased risks of colorectal, breast, and prostate cancers, with meta-analyses reporting risk reductions of 15–30% with higher vitamin D status [7,8,9]. Mechanistically, calcitriol inhibits tumor growth by promoting apoptosis, suppressing angiogenesis, and modulating the tumor microenvironment through VDR-mediated gene regulation [9,10]. In vitro and animal models further support these anti-cancer properties, showing reduced proliferation and metastasis in cancer cell lines exposed to 1,25(OH)2D [11]. However, clinical evidence remains mixed; while some trials suggest protective effects, others find no causal link, highlighting variability across cancer types, populations, and study designs [12]. This inconsistency raises a critical question: could vitamin D’s role extend to less-studied malignancies, such as those of the head and neck, where its receptor is also widely expressed [13]? 

Head and neck cancers (HNCs) encompass a heterogeneous group of malignancies originating in the oral cavity, pharynx, larynx, nasal cavity, and salivary glands, with squamous cell carcinoma (SCC) predominating, particularly in the oral cavity [14,15]. GLOBOCAN 2022 estimates rank lip and oral cavity cancers 16th globally by incidence (377,713 cases), but combining nasopharyngeal, oropharyngeal, hypopharyngeal, and salivary gland cancers elevates HNC to approximately 8th, with over 650,000 cases annually [16,17]. This burden is rising, driven by multifactorial etiologies; tobacco and excessive alcohol use synergistically increase risk by up to five-fold, while environmental carcinogens and viral infections—human papillomavirus (HPV) in oropharyngeal cancers and Epstein–Barr virus (EBV) in nasopharyngeal cases—further complicate the landscape [18]. HPV-related oropharyngeal cancers are surging in Western countries, shifting HNC’s demographic profile [19]. In developing nations, incidence is climbing due to lifestyle changes and limited prevention, amplifying its public health toll [20]. Despite advances in surgery, radiotherapy, chemotherapy, and immune checkpoint inhibitors, survival remains stubbornly stagnant—global deaths dropped only modestly from 404,000 in 2012 to 378,785 in 2022 [21]. This persistent challenge underscores an urgent need to explore modifiable biological factors beyond traditional risk profiles.

Within this context, vitamin D emerges as a candidate warranting scrutiny in HNC. Its receptor’s expression in head and neck tissues—the salivary glands, tongue, and lymphoid cells—mirrors sites of HNC origin, hinting at a potential role in pathogenesis and prognosis [22]. Yet, evidence remains fragmented; while some studies [23] report no causal link between 25(OH)D and oral/oropharyngeal cancer risk, others [24] suggest associations with reduced incidence and improved outcomes, particularly in specific subtypes. This ambiguity, coupled with HNC’s rising burden and static survival rates, justifies a deeper dive into vitamin D’s clinical impact. Our systematic review aims to synthesize recent evidence from epidemiological studies, clinical trials, and patient-centered research, complemented by key in vitro and animal findings, to evaluate whether vitamin D status influences HNC risk, prognosis, treatment response, and complications. By addressing this gap, we seek to clarify vitamin D’s potential as a modifiable factor in HNC management, offering insights for future research and therapeutic strategies.

## 2. Materials and Methods

### 2.1. Search Strategy

**Figure 1:**
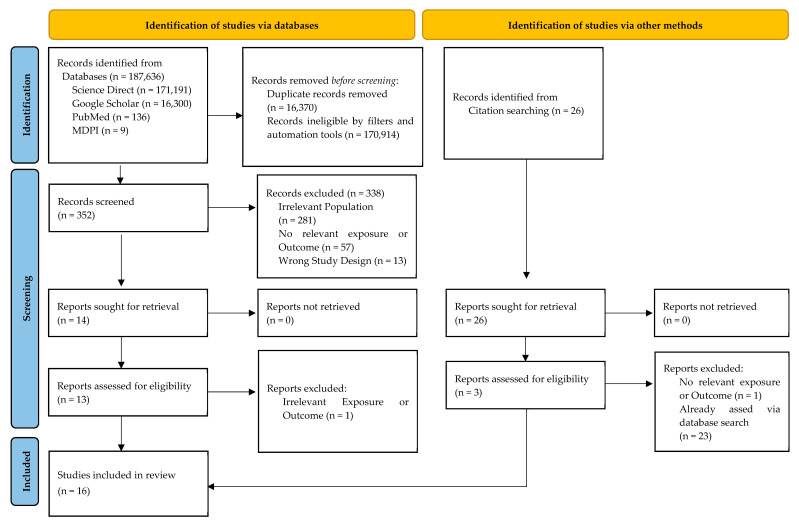
PRISMA flowchart of study selection process.

This systematic review utilized standard operating principles as outlined in the Preferred Reporting Items for Systematic reviews and Meta-Analyses (PRISMA) reporting guidelines [25] (Figure 1).

Databases included PubMed, Google Scholar, and ScienceDirect, supplemented by searches in MDPI journals, to overcome query limitations like ScienceDirect’s Boolean operator cap.

The search strategy involved the use of specific keywords and phrases, utilizing the MeSH feature of PubMed, such as “head and neck cancer”, “Oral Cancer”, “Vitamin D”, “25(OH)D3” “Cholecalciferol”, “Calcitriol”, “Squamous Cell Carcinoma of Head and Neck”, “Oropharyngeal Neoplasms”, “Laryngeal Neoplasms”, “Survival Rate”, “Treatment Outcome”, “Prognosis”, and “VDR”, and Boolean Operators such as AND, OR, and NOT were used to refine search results (e.g., (“Vitamin D”[MeSH] OR “Cholecalciferol”[MeSH]) AND (“Head and Neck Neoplasms”[MeSH] OR “Squamous Cell Carcinoma of Head and Neck”[MeSH])).

Articles available in the English language were used, and the rest were excluded. Abstracts were reviewed to select studies that met the inclusion criteria. Only articles that had the full text available were utilized for review and citation. Any duplicates from different databases and various search queries were then removed. When possible, the search was narrowed down by excluding paper categories other than research articles, such as book chapters, correspondence, editorials, mini reviews, etc.

We employed a snowballing citation search method by systematically examining the reference lists of key articles identified during the initial search. By tracing previous works’ notable citations, we aimed to identify highly relevant publications that may not have been captured in our primary search strategy and to thus yield a more complete literature review. Using a few key articles as “seed” papers and expanding the search based on their citations and indexing terms also helped in broadening the literature review.

Our review included publications from the last 10 years to ensure that the most current and relevant research was considered. However, through the snowballing method, highly cited papers were sought, with the aim of consulting older yet fundamental papers for the topic in question. This review was not prospectively registered in any database.

### 2.2. Study Selection and Data Analysis

To ensure the quality and reliability of the included sources, two authors independently assessed the publications. Any disagreements were resolved through discussion or, when necessary, by consulting a third author. For the screening process, two independent reviewers (A.A. and C.P.B.) evaluated all records for eligibility. The inter-rater reliability, measured by Cohen’s Kappa, was 0.81, indicating a high level of agreement. Discrepancies were addressed through consensus or, if unresolved, by involving a third reviewer (B.H).

The inclusion criteria were (1) studies with abstracts relevant to the potential role of vitamin D in the evolution of and response to the treatment of head and neck cancers; (2) full-text articles published in English from 1 January 2015 to 1 March 2025; and (3) original research articles with a given study design (human studies (observational cohort studies and controlled trials) with consideration given to animal and in vitro studies in the Introduction and Discussion sections).

The exclusion criteria were (1) studies focusing on topics irrelevant to our research objective; (2) articles published in a language other than English, with no translation available; (3) studies not published in a peer-reviewed journal; (4) research resources without available full-text (abstract only); and (5) inappropriate study designs (e.g., letters, case reports, conference abstracts, etc.).

After the final list of selected studies was agreed upon, two separate reviewers (B.H. and E.C.) independently extracted the relevant data from the included studies and systematically organized it into a table, ensuring a methodological approach for our analysis. The following data were extracted: study ID (first author and year of publication), study design, sample size, sample type, analysis methodology, response to treatment, and statistical significance. Any gaps in the data presented in selected studies were addressed as weaknesses when discussing said studies.

When presenting results, the statistical measures used to report outcomes were as follows: odds ratio (OR) used to assess the association between vitamin D levels and HNC risk; hazard ratio (HR) used for survival analysis, evaluating mortality risk, and overall survival; mean and median differences used to compare vitamin D levels between HNC patients and controls; and *p*-values used to assess statistical significance. Studies selected for each synthesis were based on whether the data in question were present or not. Due to heterogeneity in data across studies, missing data were not handled in any specific manner.

## 3. Results

### 3.1. Overview of Selected Studies

The initial search, using keyword variants in advanced searches, identified 187,642 records addressing vitamin D, head and neck cancer (HNC), other cancers, and diverse populations. This broad yield stemmed from ScienceDirect’s eight-operator limit, necessitating multiple queries, while PubMed, Google Scholar, and MDPI journals allowed refined searches. A 10-year timeframe (2015–2025) ensured inclusion of current research, reflecting data up to March 2025. Before screening, 170,914 records were excluded, primarily via filters (e.g., language, study type) and automation tools targeting relevant samples. After consolidating results across databases and queries, 16,370 duplicates were removed, leaving 352 papers for abstract screening. Of these, 338 were excluded, and 14 underwent full-text review, with 13 deemed eligible. Via snowballing citation searching, 3 additional papers were added, resulting in 16 studies included. This selection process is detailed in Figure 1.

Participant ages varied, from a mean of 42.67 ± 10.83 years [26] to a median of 69 years (range 60–78) [27], with most studies targeting middle-aged adults (40–60 years). Several included matched controls, who were primarily healthy individuals, for serum 25(OH)D comparisons [26,28,29,30]. The studies spanned diverse geographic regions and ethnic groups, with higher deficiency suggested in non-Caucasian and lower socioeconomic groups in some contexts [30,31]. HNC patients consistently showed lower 25(OH)D levels than controls, supporting a potential link to cancer incidence and progression [26,28,29,30]. Deficiency prevalence ranged from 47% to 95%, varying by population and subtype [30,32], with severe deficiencies noted in advanced-stage, malnourished, or intensively treated patients [29,31,32,33].

Table 1 provides a detailed overview of the studies included in this review, highlighting study designs, populations with HNC, clinical outcomes, and key findings.

### 3.2. Prevalence of Vitamin D Deficiency in Head and Neck Cancer Patients

Among the included studies, six reported a high prevalence of vitamin D deficiency in patients with head and neck cancer [28,29,30,31,33,40]. The prevalence of vitamin D deficiency in these studies is summarized in Table 2. Both Fanidi et al. and Ulaganathan et al. reported some high rates of vitamin D deficiency in HNC patients [28,30]. Approximately 75% of cases had vitamin D levels below 20 ng/mL, and 95% of cases showed vitamin D deficiency, suggesting that low vitamin D levels may be a common risk factor for nasopharyngeal carcinoma (NPC) [28]. Similarly, Bochen et al. reported that 47% of patients had vitamin D deficiency [29], a finding that was mirrored by Radivojevic et al., who observed that approximately 47% of their study population had deficient levels [33]. Additionally, Kapala et al. found that 66.8% of cancer patients were deficient in vitamin D, even among those receiving supplementation, suggesting that standard vitamin D supplementation may not be sufficient for these patients [30]. Bhanu et al. further reinforced this issue, reporting that 71.42% of patients had suboptimal vitamin D levels [40].

### 3.3. Influence of Demographic Characteristics and Season

Among the included studies, some did not address the variations between 25(OH)D levels and demographic characteristics [26,27,30,32,33,34,39,40,42]. This is not to be confused with the fact that the included studies did not contain the demographic characteristics of the study groups, but no stratification by 25(OH)D status was performed, rather stratifications by existing condition (cancer/cancer-free), progression, intervention, survival, and/or other correlations with demographic characteristics were performed instead.

Pu et al. suggests a possible negative correlation between age and vitamin D, but in no conclusive manner due to possible bias, since HNC is highly prevalent around 50 years old [36]. This finding is supported, however, by the work of Kapała et al., which shows increased vitamin D deficiency with age. Their findings also include a significantly higher occurrence of vitamin D deficiency in males (*p* < 0.001) [31].

Fanidi et al. observed that current smokers had 7% lower 25(OH)D concentrations on average (95% CI: −14% to 0%), while former smokers had 9% higher 25(OH)D concentrations (95% CI: 2–16%) and seasonal variation, with the lowest levels among patients tested around February and March and the highest levels among patients tested in August. BMI was inversely associated with 25(OH)D3 concentrations [28].

Bochen et al., however, concluded that vitamin D deficiency is no indicator of a general malnutrition status, as neither BMI nor serum albumin level were associated with 25(OH)D levels [29].

Both Westmark et al. and Fanidi et al. showed no differences in 25(OH)D level in relation to alcohol intake [28,37].

### 3.4. Impact of Vitamin D on Cancer Risk and Development

Four studies showed a significant association between low vitamin D levels and an increased risk of HNC. Both Fanidi et al. and Vaughan-Shaw et al. demonstrated a significant inverse association between vitamin D levels and HNC risk [28,34]. Fanidi et al. reported that a doubling of 25(OH)D levels was associated with a 30% lower risk of HNC (OR = 0.70, 95% CI: 0.56–0.88, *p*-trend = 0.001), with even stronger associations observed in specific subtypes such as laryngeal and hypopharyngeal cancer (OR = 0.55, 95% CI: 0.39–0.78). Vaughan-Shaw et al. supported these findings, showing that higher vitamin D levels were associated with a 32% reduction in HNC risk (HR = 0.74, 95% CI: 0.66–0.82). Similarly, Pu et al. and Ulaganathan et al. found that higher vitamin D intake and serum levels were protective against HNC [30,36].

Pu et al. conducted a large-scale analysis of 81,908 participants and found that higher vitamin D intake was linked to a significantly lower incidence of HNC (OR = 0.68, 95% CI: 0.59–0.78). Ulaganathan et al. focused on nasopharyngeal carcinoma (NPC) and reported that lower vitamin D levels were associated with an increased NPC risk (AOR = 0.73, 95% CI: 0.57–0.94, *p* = 0.016), further supporting the hypothesis that vitamin D deficiency may contribute to cancer development. These findings are illustrated in Table 3.

A noteworthy consideration when analyzing these results summarized in Table 3 that two studies (Vaughan-Shaw et al., and Pu et al.) are meta-analyses, and they present condensed comparisons of previous research. The results are in accordance with the more recent findings of Ulaganathan et al., 2024, as presented.

### 3.5. Vitamin D and Prognostic Outcomes

Among the nine studies that examined clinical outcomes [27,28,29,33,34,35,36,37,40], eight found a positive association between higher vitamin D levels and improved survival, though statistical significance was not always reached, while three studies examined its role in recurrence and disease progression [29,35,36]. Their findings are summarized in Table 4. Fanidi et al. found that each doubling of 25(OH)D levels reduced mortality risk by 27% (HR = 0.73, 95% CI 0.55–0.97). Patients with vitamin D levels of 10 ng/mL had a 1.72-fold higher risk of death compared to those with 20 ng/mL, but no additional survival benefits were observed above this threshold [28]. Similarly, Vaughan-Shaw et al. reported that higher vitamin D levels were associated with better overall survival (HR = 0.74, 95% CI: 0.66–0.82) and progression-free survival (HR = 0.84, 95% CI: 0.77–0.91) [34].

In a cohort of 231 HNSCC patients, Bochen et al. found that low vitamin D levels were linked to shorter overall survival, with 42.6% of patients in the vitamin D-deficient group dying compared to 30.3% in the sufficient group (*p* = 0.0085). The study also found that vitamin D levels significantly influenced survival in HPV-negative patients (*p* = 0.018) but had no effect in HPV-positive cases (*p* = 0.98) [29]. Similarly, Pu et al. demonstrated that higher vitamin D levels correlated with lower HNC mortality (HR = 0.75, 95% CI 0.60–0.94) over an 8–12-year follow-up [36]. Further analyses showed that patients with higher circulating vitamin D had significantly better survival over 4–5 years (HR = 1.13, 95% CI 1.05–1.22).

Beyond survival, three studies investigated vitamin D’s role in recurrence and disease progression. Yokosawa et al. highlighted a significant inverse association between vitamin D intake and cancer recurrence (HR = 0.47, 95% CI = 0.20–1.10, *p*-trend = 0.048), with the strongest effect observed in stage 4 patients. However, no association was found between vitamin D intake and overall or HNC-specific mortality [35]. Similarly, Radivojevic et al. reported that vitamin D deficiency was linked to a 2-year disease-free survival (DFS) rate of 57%, compared to 60% in the insufficient group and 64% in the sufficient group (*p* = 0.497). For OS, rates were 60% in the deficient group, 75% in the insufficient group, and 71% in the sufficient group (*p* = 0.577), though none of these differences reached statistical significance [33].

### 3.6. Vitamin D and Treatment-Related Toxicity

Four studies examined the relationship between vitamin D levels and treatment-related toxicity [26,32,39,40]. Two focused on the benefits of vitamin D supplementation [26,39], while two highlighted the association between low vitamin D levels and increased toxicity rates [32,40]. Table 5 summarizes their findings with regard to treatment toxicity.

Both Anand et al. and Abdelaziz et al. demonstrated that vitamin D supplementation significantly reduced treatment-related side effects. Anand et al. found that supplementation improved chemoradiation-induced toxicity, leading to significant reductions in mucositis, pain, and swallowing difficulties (*p* < 0.001), alongside an overall enhancement in quality of life [26]. Similarly, Abdelaziz et al. reported that supplementation was associated with lower rates of oral mucositis, skin toxicity, taste changes, and dysphagia (*p* < 0.001 for mucositis), suggesting a protective effect of vitamin D in mitigating treatment-related side effects [39].

On the other hand, Nejatinamini et al. and Bhanu et al. linked low vitamin D levels to greater treatment toxicity [32,40]. Nejatinamini et al. observed that 52% of patients developed moderate to severe mucositis, and those affected had significantly lower plasma vitamin D levels compared to patients without mucositis (*p* < 0.02) [32]. Bhanu et al. further supported these findings, reporting that patients with optimal vitamin D levels had significantly lower rates of radiation-induced dermatitis and mucositis (*p* = 0.0011) [40].

### 3.7. Vitamin D, Malnutrition, and Postoperative Complications

Four studies examined the relationship between vitamin D levels, malnutrition, and postoperative complications [31,32,33,38]. Two studies focused on the impact of vitamin D deficiency on malnutrition and muscle loss [31,32], while two highlighted its predictive role in postoperative complications [33,38]. Table 6 presents the key findings of these articles.

Both Nejatinamini et al. and Kapala et al. demonstrated a strong association between low vitamin D levels and malnutrition [31,32]. Kapala et al. found that weight loss was significantly associated with vitamin D deficiency (*p* = 0.002), suggesting that maintaining adequate vitamin D levels may be essential for nutritional stability in cancer patients. Nejatinamini et al. found that patients with vitamin D deficiency had a higher risk of malnutrition (OR = 1.76, 95% CI: 1.02–3.04) and that low vitamin D levels were significantly associated with muscle loss (*p* = 0.031), suggesting a detrimental effect on nutritional status and physical function.

On the other hand, Nett et al. and Radivojevic et al. linked low vitamin D levels to increased postoperative complications [33,38]. Nett et al. found that patients with vitamin D deficiency experienced greater weight loss (pT2 = 0.0031, pT4 = 0.0424) and were at higher risk of digestive issues and muscular complaints (*p* = 0.0062 and *p* = 0.0448, respectively), reinforcing the role of vitamin D in post-surgical nutritional recovery. Radivojevic et al. further expanded on this by showing that vitamin D deficiency was a strong predictor of postoperative complications (OR = 2.4, 95% CI: 1.30–4.42, *p* = 0.011) and that patients with high malnutrition risk had significantly lower 2-year survival rates (30% vs. 62% and 83%, *p* = 0.010). Multivariate analysis confirmed both vitamin D deficiency and malnutrition as independent risk factors (*p* < 0.05), highlighting the clinical importance of vitamin D in surgical outcomes.

## 4. Discussion

Vitamin D’s role in head and neck cancer (HNC) weaves a complex narrative, one that our systematic review of 16 studies (2015–2025) sought to unravel with a rigorous, systematic approach. We found a striking prevalence of vitamin D deficiency (47–95%) among HNC patients, especially those with advanced-stage disease or those under intensive treatment, alongside a 26–32% reduced HNC risk with higher 25(OH)D levels (e.g., OR 0.68–0.74). Several studies within this review [27,28,29,33,34,36,37,41] suggest that elevated 25(OH)D levels may enhance survival outcomes in HNC patients. Particular attention has been drawn to vitamin D’s capacity to regulate immune checkpoint pathways and influence the tumor microenvironment, notably in HPV-positive HNCs, where immune modulation significantly governs disease progression.

### 4.1. Vitamin D Supplementation

The relationship between vitamin D status and clinical outcomes in HNCs is complex and influenced by multiple factors, including genetic variability in vitamin D receptor (VDR) expression, tumor heterogeneity, and patient-specific metabolic differences, all these serving as weak points in clinical studies [42,43]. Emerging evidence indicates that VDR polymorphisms may modulate treatment efficacy and prognostic trajectories [34,44,45,46], underscoring the potential for personalized therapeutic strategies involving vitamin D as an adjunct. Specifically, variants such as FokI and TaqI can alter VDR’s binding affinity to calcitriol, disrupting downstream gene regulation of apoptosis and immune response pathways critical to tumor control [46,47]. These polymorphisms may also influence VDR expression levels in HNC tissues, potentially amplifying or dampening the anti-tumor effects of vitamin D supplementation, thus shaping individual responses to therapy and survival outcomes.

Previous studies on vitamin D supplementation in head and neck cancer (HNC) offer a critical lens for contextualizing our systematic review’s findings, revealing both alignment and divergence. Our observed reductions in treatment-related toxicities, such as mucositis and dysphagia (*p* < 0.001) with supplementation [26,39], align with Ruggiero et al. (2006), who reported a 35% lower mucositis incidence (*p* = 0.03) in HNC patients given 2000 IU/day during radiotherapy [48]. However, our mixed survival outcomes—i.e., benefits in some studies (e.g., HR 0.73–0.84) [28,34,36] and none in others [26,35]—mirror the work of Wactawski-Wende et al. (2006), where 1000 IU/day provided no survival advantage across cancers (HR 0.98, 95% CI 0.91–1.05) [49]. Zarrati et al. found that high-dose vitamin D supplementation (>4000 IU/day) significantly reduced treatment-induced pain in cancer patients (SMD −0.49, *p* = 0.005) [50], corroborating our observed reductions in mucositis and dysphagia (*p* < 0.001) [26,39], suggesting a shared anti-inflammatory mechanism enhancing patient tolerance. Martinez-Alonso et al. reported that 25(OH)D levels below 20 ng/mL increased cancer mortality (HR 1.42, 95% CI 1.18–1.71) with benefits plateauing above 40 ng/mL [51], aligning with our high deficiency prevalence (47–95%) and non-linear survival trends and reinforcing the prognostic relevance of baseline vitamin D status in HNC. This high prevalence of deficiency is further corroborated by Alexandru et al., who reported that 50–90% of pediatric cancer patients exhibited 25(OH)D levels below 20 ng/mL during chemotherapy, highlighting a consistent vulnerability across cancer populations [9].

While confounding variables such as BMI, diet, alcohol consumption, tobacco exposure, race, education and economic status might seem to play a significant role at first, among the presented studies, evidence is scarce and the conclusion leans towards little to no impact of 25(OH)D levels in HNC patients [28,37]. For example, Fanidi et al. showed that even when accounting for most confounding variables (education, alcohol consumption, circulating cotinine, tobacco exposure, and BMI) existing correlations suffer little change [28].

The divergence in findings across vitamin D supplementation studies likely stems from genetic variability in the vitamin D receptor, as evidenced by polymorphisms such as FokI and TaqI, which modulate treatment efficacy and prognostic outcomes in HNC. FokI variants can impair VDR’s calcitriol-binding affinity, altering regulation of apoptosis and immune pathways [47,52], while TaqI affects mRNA stability, potentially reducing VDR expression and responsiveness to supplementation [53], thus contributing to inconsistent survival benefits. These parallels with prior work affirm vitamin D’s potential in HNC management yet underscore the need for tailored strategies to unlock consistent benefits. We recommend that supplementation be administered only after categorizing patients by deficiency status, thereby ensuring safety and maximizing therapeutic impact, rather than as a universal practice. Additionally, stratifying patient populations by VDR polymorphisms, HPV status, and baseline 25(OH)D levels could identify responders versus non-responders, refining vitamin D’s role as an adjunct in personalized HNC care.

Clinical evidence suggests that vitamin D supplementation may potentiate responses to radiotherapy and chemotherapy in head and neck cancer (HNC), yet its interactions with other treatments warrant cautious consideration. Studies like that by Abdelaziz et al. [39] within our review demonstrate that adequate 25(OH)D levels (>30 ng/mL) enhance radiotherapy efficacy, reducing the severity of oral mucositis (*p* < 0.001) by upregulating DNA repair genes (e.g., GADD45) and dampening inflammation via NF-κB suppression. These findings are based on previous work by Feldman et al., who noted calcitriol’s potentiation of cisplatin cytotoxicity in HNSCC cells [54]. Conversely, the National Cancer Institute cautions that dietary supplements like vitamin D can alter cancer treatment efficacy and safety, as evidenced by Christakos et al., who describe high-dose vitamin D inducing CYP3A4 via VDR/PXR pathways, potentially accelerating the metabolism of drugs like imatinib and increasing toxicity risk [55]. Similarly, Schwartz et al. found that 800 IU/day vitamin D2 inhibited CYP3A4, raising atorvastatin levels by 17% (*p* = 0.04) in non-cancer patients [56], suggesting bidirectional pharmacokinetic shifts that could elevate HNC drug levels (e.g., cisplatin) or reduce efficacy. These mechanisms—VDR-driven gene regulation and CYP enzyme modulation—highlight supplementation’s dual potential to amplify therapeutic benefits while posing interaction risks, urging tailored monitoring in HNC care.

### 4.2. Limitations and Future Research Suggestions

This systematic review acknowledges several limitations inherent in the synthesized literature, including inconsistencies across study designs, the scarcity of large-scale randomized controlled trials (RCTs), and potential confounding factors such as lifestyle variables, nutritional status, and comorbidities, all of which may obscure the true impact of vitamin D on HNC outcomes. Our decision to include only studies from the past decade (2015–2025), while ensuring relevance and alignment with current clinical and research standards, may have overlooked valuable older investigations; however, this temporal constraint also minimized noise from outdated findings, with key foundational studies retained to anchor the analysis. The exclusion of non-English-language studies introduced a regional bias, potentially underrepresenting data from high-prevalence HNC areas, while the systematic selection process, though rigorous, remains susceptible to human judgment errors. Variability in study designs, patient cohorts, and vitamin D assessment methods—ranging from serum thresholds to timing—fostered heterogeneity, challenging the generalizability of our conclusions. Additionally, the limited availability of data on specific clinical endpoints—such as infection rates, recurrence beyond two years, or quality of life (QoL) metrics—constrained our ability to link vitamin D levels directly to tangible patient outcomes, potentially diminishing real-world applicability. Furthermore, the absence of uniform supplementation protocols across studies complicates direct comparisons, and our reliance on observational data over interventional evidence limits causal inference, leaving gaps in understanding optimal therapeutic strategies.

To address these shortcomings, well-designed, large-scale randomized controlled trials are imperative to rigorously evaluate vitamin D supplementation’s efficacy in improving HNC outcomes, with meticulous monitoring to manage potential toxicity risks and drug interactions. Standardizing supplementation protocols, establishing consistent deficiency thresholds (e.g., <20 ng/mL), and defining precise measurement timepoints represent critical initial steps to enhance the quality of future studies. It is also worth comparing the efficacy of dietary changes versus supplementation, as current evidence is insufficient.

Moreover, investigations should explore the interplay between vitamin D supplementation and VDR polymorphisms, assessing their combined influence on survival, recurrence, and QoL across treatment stages and chemotherapeutic agents. Incorporating additional biomarkers—such as parathyroid hormone, vitamin D-binding protein, oxidative stress markers, or interleukins—could deepen insights into vitamin D’s mechanistic role, while its potential as a preventive intervention also merits attention. Examining geographic and ethnic variations in deficiency prevalence, alongside long-term effects on survivors’ bone health, immune function, and QoL, will pave the way for more personalized HNC treatment approaches, tailoring interventions to individual patient profiles and disease contexts.

## 5. Conclusions

This systematic review elucidates the multifaceted role of vitamin D status in head and neck cancer (HNC), synthesizing evidence from 16 studies (2015–2025) to highlight its impact on risk, prognosis, treatment outcomes, and complications. We identified a high prevalence of vitamin D deficiency (47–95%) among HNC patients, which was particularly pronounced in advanced-stage disease and intensive treatment settings, alongside a consistent 26–32% reduction in HNC risk with higher 25(OH)D levels (OR 0.68–0.74). Adequate vitamin D levels correlated with improved survival in several studies (HR 0.73–0.84), though benefits plateaued beyond a threshold (~20 ng/mL), suggesting a non-linear relationship, while supplementation significantly mitigated treatment-related toxicities, such as mucositis and dysphagia (*p* < 0.001), thus enhancing patient tolerance. Conversely, deficiencies exacerbated malnutrition and postoperative complications (OR 2.4), underscoring vitamin D’s broader clinical relevance. These findings align with prior research affirming vitamin D’s therapeutic potential, but its variability—potentially driven by VDR polymorphisms (e.g., FokI, TaqI)—and interactions with treatments like imatinib highlight the need for precision. We advocate for routine 25(OH)D screening to identify deficient HNC patients (<20 ng/mL) for targeted supplementation, coupled with stratification by VDR genotypes, HPV status, and baseline levels in order to optimize efficacy and safety. While these insights position vitamin D as a modifiable factor in HNC management, the heterogeneity and observational nature of the evidence necessitate large-scale RCTs to confirm causality and refine strategies, paving the way for personalized interventions that could meaningfully alleviate HNC’s burden.

## Figures and Tables

**Table 1:** Overview of the included studies: HC—healthy controls; NM—not mentioned, HNC—head and neck cancer; CI—confidence interval; OR—odds ratio; HR— hazard ratio; QOL—quality of life; NA—not applicable OS—overall survival, PFS—progression-free survival; OPC—oropharyngeal cancer; * median age; ^1^ no stratification by type of cancer for demographic characteristics, only the whole study group.

Study, Year	Study Design	Age, Mean/Median *	HNC Population	Control Population	Definition of Vitamin D Deficiency	Clinical Outcome Assessed	Key Findings	Conclusions
Fanidi et al., 2016 [28]	Nested case–control study within EPIC cohort	56.7 years * (42–71)	350	497 matched HC and 443 unmatched HC	NM	HNC incidence, mortality, survival rates	A doubling of 25(OH) levels was associated with reduced HNC risk (OR 0.70, 95% CI 0.56–0.88). Inverse association strongest for larynx/hypopharynx (OR 0.55) and oral cavity (OR 0.60). No clear association with oropharynx cancer. Lower 25(OH)D3 linked to increased mortality in HNC (HR 0.73). Doubling in plasma of 25(OH)D3 associated with 45% lower risk.	Higher pre-diagnostic vitamin D levels linked to lower HNC risk and improved survival. Association not explained entirely by smoking, alcohol, or BMI. Findings suggest a potential protective role of vitamin D.
Anand et al., 2017 [26]	Prospective observational study	42.67 ± 10.83 years	87	95 HC	Deficiency < 20 ng/mL, Insufficiency 21–29 ng/mL	Treatment-related toxicity, QOL, oral mucositis, swallowing performance	Vitamin D supplementation reduced therapy-related toxicities and improved QOL in advanced cancer patients.	Vitamin D deficiency is prevalent among patients. Supplementation helps in reducing treatment-related toxicities and improving QOL, especially in advanced cancer.
Vaughan-Shaw et al., 2017 [34]	Systematic review and meta-analysis	NM	628	NA	NM	OS, PFS, cancer-specific mortality, disease progression	An increased survival rate, but not significant and PFS (HR 0.84, 95% CI 0.77–0.91).	Higher vitamin D levels correlate with better cancer prognosis.
Bochen et al., 2018 [29]	Prospective observational study	63 years *	231	232 HC	Deficiency < 10 ng/mL, Insufficiency 10–30 ng/mL	OS, lymph node metastasis, HPV status	HNSCC patients had significantly lower vitamin D levels than controls (median: 11.1 ng/mL vs. 21.8 ng/mL). Low vitamin D levels were associated with lymphatic metastasis and poor OS. HPV-positive patients have higher vitamin D levels. Low vitamin D serum levels correlated with significantly shorter OS in HPV-negative patients (*p* = 0.018).	Vitamin D deficiency is prevalent in HNSCC patients and predicts worse survival. Vitamin D may enhance immune response and could be beneficial as an adjunct in immunotherapy.
Nejatinamini et al., 2018 [32]	Prospective cohort study	60.3 ± 10.8 years	28	NA	Deficiency * < 20 ng/mL, Insufficiency * 20–30 ng/mL	Skeletal muscle loss, mucositis severity, inflammation, body composition changes	Vitamin D deficiency was prevalent in HNC patients, with plasma 25(OH)D levels remaining stable during treatment despite increased dietary intake. Patients with lower vitamin D levels experienced greater skeletal muscle loss. Mucositis was more common in patients with lower plasma 25(OH)D.	Vitamin D deficiency may contribute to greater muscle loss and higher mucositis severity in HNC patients. Assessing and correcting vitamin D levels before cancer treatment could help reduce treatment-induced toxicities.
Weinstein et al., 2018 [27]	Prospective cohort study	69 years ^1^ (60–78)	398	NA	NM	Overall cancer mortality, site-specific survival (HNC, prostate, kidney, melanoma, lung), stage-specific survival, stratified analyses by smoking and BMI	Higher 25(OH)D levels were associated with lower overall cancer mortality (HR = 0.76, 95% CI 0.67–0.85, *p* < 0.0001). For head and neck cancer, higher 25(OH)D showed a trend towards improved survival but was not statistically significant.	Higher vitamin D levels prior to cancer diagnosis are linked to better survival outcomes for many cancers, including a trend for HNC. Findings suggest potential benefits of maintaining adequate vitamin D levels before cancer development.
Yokosawa et al., 2018 [35]	Prospective cohort study	<200: 58.6 ± 12.7 years [200–465): 58.5 ± 11.1 years [465–675): 60.0 ± 9.5 years ≥675: 63.1 ± 10.4 years	434	NA	NM	OS, recurrence rates, stratified analyses by stage, sex, BMI	Higher total vitamin D intake was associated with lower recurrence risk (Q4 vs. Q1 HR = 0.47, 95% CI = 0.20–1.10, *p*-trend = 0.048), especially in individuals with advanced cancer. No significant association was observed with overall survival or HNC-specific survival.	Findings suggest that higher vitamin D intake may reduce recurrence risk in HNC patients. Future research should explore supplementation as a potential intervention for recurrence prevention.
Kapala et al., 2021 [31]	Cross-sectional observational study	NM	90	NA	Deficiency < 30 ng/mL, Very Low < 20 ng/mL, Optimal 30–50 ng/mL, Toxic > 100 ng/mL	Prevalence of vitamin D deficiency, correlation with cancer type, influence on weight loss, recommended supplementation dose	Vitamin D deficiency was diagnosed in 76.7% of HNC patients. Head and neck cancer patients were more likely to be vitamin D-deficient than breast cancer patients. Low vitamin D levels were associated with greater weight loss.	Vitamin D deficiency is highly prevalent in cancer patients, particularly those with head and neck cancer. Daily supplementation of at least 2250 IU is recommended to prevent deficiency, which is higher than standard guidelines.
Pu et al., 2021 [36]	Systematic review and meta-analysis	NM	81,908	NA	NM	HNC incidence, HNC-specific mortality, overall survival	Higher circulating 25(OH)D levels reduced HNC incidence by 32% (OR = 0.68, 95% CI = 0.59–0.78). Vitamin D intake was associated with a lower HNC risk (OR = 0.77, 95% CI = 0.65–0.92). Higher vitamin D levels improved survival (HR = 1.13, 95% CI = 1.05–1.22) over 4–5 years.	Higher vitamin D levels may protect against HNC incidence and improve prognosis. Findings highlight the need for further trials on vitamin D supplementation in HNC patients.
Westmark et al., 2021 [37]	Retrospective observational	59 years * (27– 87)	81	NA	Insufficiency < 30 ng/mL, Sufficiency ≥ 30 ng/mL	Association with HPV status, OS, prognostic significance of vitamin D levels	No significant association between vitamin D levels and HPV status (*p* = 0.354). Survival probability at 2 years: 88% in sufficient vitamin D group vs. 74% in insufficient group (*p* = 0.1675). There was a trend towards poorer survival in vitamin D-insufficient patients, but it was not statistically significant.	Vitamin D status may be a prognostic factor in OPC, though results were not statistically significant. Further research is needed to explore the immune-mediated role of vitamin D in HPV-related OPC.
Nett et al., 2022 [38]	Prospective randomized comparative trial	63.55 ± 13.61 years	22	24	NM	Weight loss, BMI stability, nutritional deficiencies, postoperative digestive and muscular complaints, QOL, physical activity	Patients in the intervention group had significantly less weight loss (pT2 = 0.0031, pT4 = 0.0424), better BMI stability (pT2 = 0.0496). They reported significantly fewer digestive problems (*p* = 0.0062) and muscular complaints (*p* = 0.0448), better dietary habits (*p* = 0.0348), and higher physical activity levels (*p* = 0.0045) compared to controls.	Postoperative nutritional intervention, including professional counseling and vitamin supplementation, reduces weight loss and improves nutritional status, quality of life, and physical activity. Vitamin D supplementation, along with protein, zinc, and calcium, is recommended postoperatively.
Abdelaziz et al., 2024 [39]	Prospective case–control study	57.12 ± 11.79 years	30	31	Deficiency < 20 ng/mL, Insufficiency 21–29 ng/mL, Sufficiency 30–100 ng/mL	Oral mucositis severity, skin toxicity, dysphagia, taste changes, xerostomia, treatment response, radiotherapy-related toxicity	Vitamin D supplementation significantly reduced oral mucositis (*p* < 0.001 in weeks two to six). Skin toxicity, taste changes, and dysphagia improved significantly in the vitamin D group. There was a higher incidence of xerostomia in the vitamin D group in weeks six and seven, though not statistically significant. Vitamin D supplementation improved radiotherapy response and reduced treatment interruptions.	Vitamin D administration during radiotherapy reduces oral mucositis, improves treatment tolerance, and enhances response rates. Vitamin D has potential as an adjunct therapy for mitigating radiotherapy-induced toxicities in HNC patients.
Bhanu et al., 2024 [40]	Prospective longitudinal study	57 years * (24–86)	28	NA	Optimal * > 30 ng/mL, Suboptimal * ≤ 3 0 ng/mL	Oral mucositis, radiation dermatitis, hemoglobin levels, white blood cell counts, treatment response	Suboptimal vitamin D levels were associated with significantly higher mucosal toxicities (*p* = 0.0011) and radiation dermatitis (*p* = 0.0505). There were no significant differences in hemoglobin or total leukocyte count between optimal and suboptimal groups. Some 71.43% of patients had a complete response, with no significant differences between groups.	Lower 25(OH)D levels correlated with increased radiation toxicities in HNSCC patients. Future studies are needed to explore the potential of vitamin D supplementation in improving treatment outcomes.
Brust et al., 2024 [41]	Retrospective observational study	64.2 years *	116	NA	Serum 25(OH)D categorized as Vit D-low (≤10 ng/mL) and Vit D-high (>10 ng/mL)	HPV status correlation, OS	No significant correlation between Vitamin D levels and OS or biomarker expression.	Vitamin D levels showed no direct impact on patient outcomes or biomarker expression.
Ulaganathan et al., 2024 [30]	Case–control study	54.06 ± 10.94 years	300	300 matched controls	Inadequate < 30 ng/mL	NPC risk, survival rate, association with lifestyle factors (BMI, smoking, alcohol, diet)	Mean serum 25(OH)D levels were significantly lower in NPC cases (63.17 ± 19.15 nmol/L) than in controls (67.34 ± 23.06 nmol/L) (*p* = 0.016). Higher 25(OH)D levels were associated with reduced NPC risk (AOR = 0.73, 95% CI = 0.57–0.94, *p* = 0.016). Lower vitamin D status was associated with multiple NPC risk factors, including low BMI and high salted fish consumption.	Vitamin D deficiency is independently associated with increased NPC risk. Future prospective studies and randomized controlled trials are needed to confirm the role of vitamin D in NPC prevention and prognosis.
Radivojevic et al., 2025 [33]	Prospective cohort study	63.9 ± 7.43 years	64	NA	Deficiency < 30 nmol/L, Insufficiency 30–50 nmol/L, Sufficiency > 50 nmol/L	Postoperative complications, infection risk, nutrition risk index, Malnutrition Universal Screening Tool scores, two-year OS, disease-free survival	Some 47% of patients exhibited vitamin D deficiency, and 31% had insufficiency. Lower vitamin D levels were associated with increased risk of postoperative infections and complications. Patients with high nutrition risk had significantly lower two-year OS (30%) compared to medium (62%) and low-risk groups (83%) (*p* = 0.010). Vitamin D levels correlated with inflammation markers, including neutrophil-to-lymphocyte ratio.	Preoperative vitamin D deficiency and malnutrition increase the risk of postoperative complications and reduce survival. Routine vitamin D assessment and nutritional support should be considered in laryngeal cancer patients before surgery.

**Table 2:** Overview of vitamin D deficiency among the included studies.

Study, Year	Population	Vitamin D Deficiency Prevalence
Fanidi et al., 2016 [28]	350	Approximately 75% of the cases had vitamin D levels below 20 ng/mL *.
Bochen et al., 2018 [29]	231	Some 47% of patients had vitamin D deficiency.
Kapala et al., 2021 [31]	90	Some 66.8% of cancer patients were deficient, even with supplementation.
Bhanu et al., 2024 [40]	28	Some 71.42% of the patients had suboptimal vitamin D levels.
Ulaganathan et al., 2024 [30]	300	Approximately 95% of the cases had vitamin D deficiency.
Radivojevic et al., 2025 [33]	64	Approximately 47% had a vitamin D deficiency.

* A conversion from nmol/L to ng/mL was performed.

**Table 3:** Vitamin D and cancer risk.

Study	HNC Population	Cancer Risk
Fanidi et al., 2016 [28]	350	Doubling of 25(OH)D levels was associated with 30% lower odds of HNC (OR 0.70, 95% CI 0.56–0.88, *p*-trend = 0.001). Specific subtypes, including larynx and hypopharynx cancer, showed even stronger associations (OR 0.55, 95% CI 0.39–0.78)
Vaughan-Shaw et al., 2017 [34]	628	Higher vitamin D levels were associated with 32% lower HNC risk, with an HR of 0.74 (95% CI: 0.66–0.82).
Pu et al., 2021 [36]	81,908	Higher vitamin D intake was linked to reduced HNC incidence (OR 0.68, 95% CI 0.59–0.78).
Ulaganathan et al., 2024 [30]	300	Lower vitamin D levels were associated with increased NPC risk (AOR = 0.73, 95% CI = 0.57–0.94, *p* = 0.016).

**Table 4:** Vitamin D and clinical outcomes: HNSCC—head and neck squamous cell carcinoma; OS—overall survival; PFS—progression-free survival; HNC—head and neck cancer; * A conversion from nmol/L to ng/mL was performed.

Study	HNC Population	Clinical Outcomes
Fanidi et al., 2016 [28]	350	Higher pre-diagnostic 25(OH)D3 levels were linked to improved post-HNC survival, with each doubling of 25(OH)D3 reducing mortality risk by 27% (HR = 0.73, 95% CI 0.55–0.97). Patients with 10 ng/mL * had a 1.72-fold higher risk of death than those with 20 ng/mL *, but no further survival benefits were observed above 20 ng/mL *, and levels > 30 ng/mL * showed a potential increase in mortality risk.
Vaughan-Shaw et al., 2017 [34]	628	Higher 25OHD was associated with better OS (HR = 0.74, 95% CI: 0.66–0.82) and PFS (HR = 0.84, 95% CI: 0.77–0.91)
Bochen et al., 2018 [29]	231	HNSCC patients with low vitamin D had shorter OS, with 42.6% (66/155) dying from their tumor compared to 30.3% (23/76) in the high-vitamin D group (*p* = 0.0085). Low vitamin D serum levels were a predictor of poor OS in HPV—patients (*p* = 0.018) but did not influence HPV + patients (*p* = 0.98).
Weinstein et al., 2018 [27]	398	Higher 25(OH)D levels trend toward improved survival, but not statistically significant.
Yokosawa et al., 2018 [35]	434	No significant association was found between total vitamin D intake and overall or HNC-specific mortality. However, higher vitamin D intake was linked to a lower recurrence risk (HR = 0.47, 95% CI = 0.20–1.10, *p*-trend = 0.048), with a stronger effect in stage 4 patients. No association was observed when analyzing dietary and supplemental intake separately, and results were inconclusive in women due to small sample size. BMI did not modify the relationship (*p*-interaction = 0.95).
Pu et al., 2021 [36]	81,908	Higher 25(OH)D levels were associated with lower HNC mortality, with an HR of 0.75 (95% CI 0.60–0.94) over 8–12 years of follow-up. Sensitivity analyses confirmed this association, and patients with higher circulating 25(OH)D had significantly better survival over 4–5 years compared to those with lower levels, with an HR of 1.13 (95% CI 1.05–1.22), based on a fixed-effects model.
Westmark et al., 2021 [37]	81	Although not statistically significant, patients with sufficient vitamin D had higher 2-year survival (88%) vs. insufficient levels (74%, *p* = 0.1675). Similarly, HPV+ patients had better survival at 400 days (98%) compared to HPV-patients (80%, *p* = 0.1954), but no survival difference was seen within the vitamin D-insufficient group (*p* = 0.7219).
Brust et al., 2024 [41]	116	Higher vitamin D levels were associated with a trend toward improved survival, especially within the first 24 months post-diagnosis; the results did not reach statistical significance (*p* = 0.2).
Radivojevic et al., 2025 [33]	64	Vitamin D deficiency was linked to a 2-year DFS rate of 57%, compared to 60% in the insufficient group and 64% in the sufficient group (*p* = 0.497). Similarly, for OS, rates were 60% in the deficient group, 75% in the insufficient group, and 71% in the sufficient group (*p* = 0.577), though the differences were not statistically significant.

**Table 5:** Association between vitamin D levels, supplementation and treatment-related toxicity.

Study	HNC Population	Treatment Toxicity
Anand et al., 2017 [26]	87	Reports that vitamin D supplementation improved chemoradiation-induced toxicity, including mucositis and pain scores, with significant improvements in swallowing performance (*p* < 0.001) and overall quality of life
Nejatinamini et al., 2018 [32]	28	Some 52% of patients developed moderate to severe mucositis. Those with mucositis had significantly lower plasma vitamin D levels compared to those without mucositis (*p* < 0.02)
Abdelaziz et al., 2024 [39]	61	Supplementation significantly reduced oral mucositis, skin toxicity, taste changes, and dysphagia (*p* < 0.001 for mucositis)
Bhanu et al., 2024 [40]	28	Patients with optimal vitamin D levels had lower rates of radiation-induced dermatitis and mucositis (*p* = 0.0011 for mucositis)

**Table 6:** Malnutrition and postoperative complications in relation to vitamin D levels.

Study	Population	Key Findings
Nejatinamini et al., 2018 [32]	28	Low vitamin D levels were significantly associated with muscle loss (*p* = 0.031). Patients with vitamin D deficiency had a higher risk of malnutrition (OR = 1.76, 95% CI: 1.02–3.04).
Kapala et al., 2021 [31]	90	Weight loss was significantly associated with vitamin D deficiency (*p* = 0.002).
Nett et al., 2022 [38]	62	Vitamin D deficiency was linked to increased weight loss (pT2 = 0.0031, pT4 = 0.0424). Vitamin D supplementation improved albumin levels (pT2 = 0.0265). Malnourished patients had a higher risk of digestive problems and muscular complaints (*p* = 0.0062 and *p* = 0.0448, respectively).
Radivojevic et al., 2025 [33]	64	Vitamin D deficiency was predictive of postoperative complications (OR = 2.4, 95% CI: 1.30–4.42, *p* = 0.011). Patients with high malnutrition risk had significantly lower 2-year survival rates (30% vs. 62% and 83%, *p* = 0.010). Multivariate analysis confirmed vitamin D and malnutrition as independent risk factors (*p* < 0.05).

## Data Availability

The original contributions presented in the study are included in the article. Further inquiries can be directed to the corresponding author.

## Abbreviations

The following abbreviations are used in this manuscript:

HNC	Head and neck cancer
VDR	Vitamin D receptor
